# Choroid plexus volume as a proxy of neuroinflammation in depression

**DOI:** 10.1192/j.eurpsy.2023.562

**Published:** 2023-07-19

**Authors:** B. Bravi, E. Melloni, L. Servidio, E. Agnoletto, M. Paolini, S. Poletti, C. Lorenzi, C. Colombo, F. Benedetti

**Affiliations:** ^1^Division of Neuroscience, Psychiatry and Clinical Psychobiology, IRCCS San Raffaele Hospital, Milan; ^2^Vita-Salute San Raffaele University, Milan; ^3^Mood disorder Unit, IRCCS San Raffaele Hospital, Milan

## Abstract

**Introduction:**

Choroid plexus (CP) is a physiological barrier, producing cerebrospinal fluid (CSF), neurotrophic, and inflammatory factors. It’s also involved in the neuro-immune axis, facilitating the interplay between central and peripheral inflammation, allowing trafficking of immune cells. Coherently, CP enlargement has been found in psychiatric diseases characterized by inflammatory signature. Although CP volume correlates with central microglia activation in major depressive disorder (MDD), it’s never been directly associated with peripheral markers in mood disorders.

**Objectives:**

Examine CP volume in mood disorders and healthy controls (HC) in relation to clinical features and peripheral inflammatory markers.

**Methods:**

CP volume was extracted with FreeSurfer in 72 HC and 152 age- and sex-matched depressed patients: 79 BD and 73 MDD. Plasma analytes in patients were collected through immunoassay technology (Bioplex). We tested for the effect of age by group on CP volume. Then we focused on the interaction between illness duration and diagnosis in predicting CP volume. After testing the effect of specific analytes by diagnosis, we calculated moderated moderation models (SPSS, PROCESS) setting each analyte as independent variable, CP volume as predicted variable and illness duration and diagnosis as moderators. We get the effects’ significance with the likelihood ratio statistic, always controlling for age, sex, and intracranial volume.

**Results:**

Patients were comparable in illness duration and severity. CP volume is differentially distributed through groups (right: p=0.04; left: p<0.01), with higher volumes in the clinical groups. Age by group significantly predict right CP volume (p=0.01). Also, duration of illness differently predicts right CP volume in MDD and BD (p=0.03) (Figure1). Then, given the significant interaction effect of IL13 (p=0.02) and IL1ra (p=0.01) in predicting right CP, we run the moderated moderation model. Longer illness duration has an effect in strengthening the opposite predicting value of IL1ra (ΔR^2^=0.03, p<0.01) on right CP volume in MDD and BD (Figure2).

**Image:**

**Figure:**
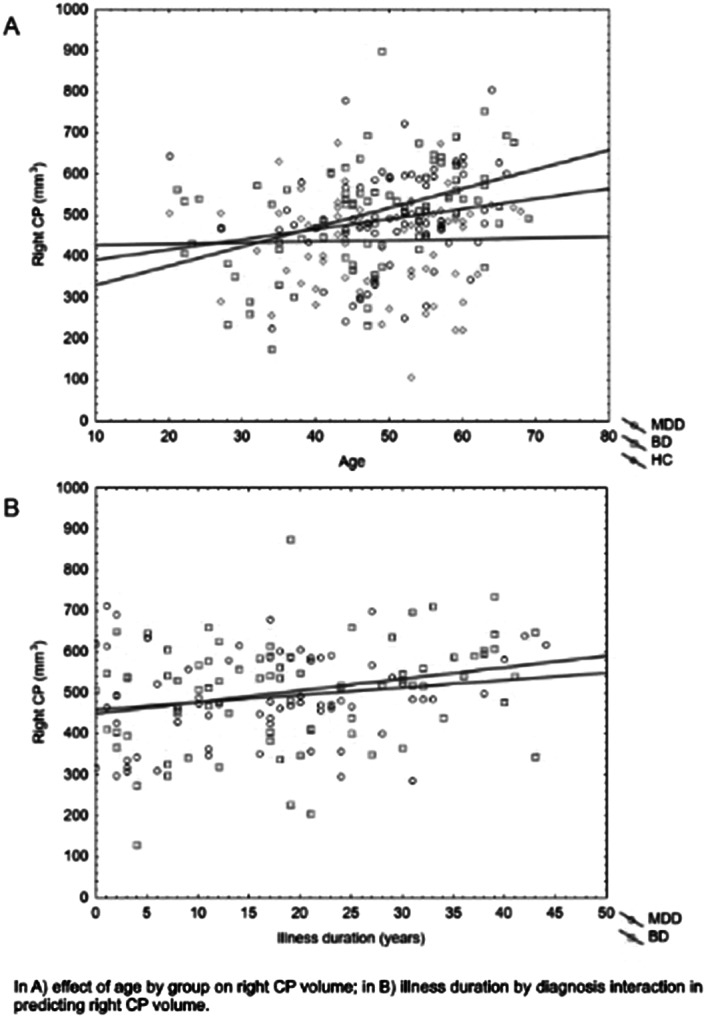

**Image 2:**

**Figure:**
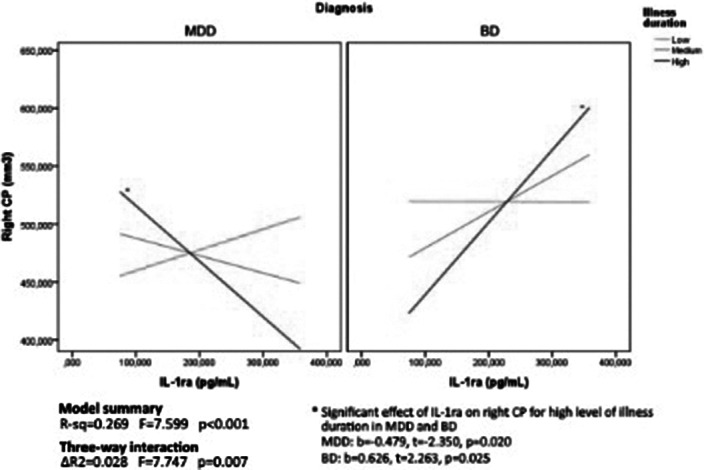

**Conclusions:**

Our findings propose CP as a proxy of inflammation in depression, being significantly predicted by peripheral immune markers in MDD and BD. In particular, the signature of inflammation in depression, could represent the neurotoxic load of the disease over the illness, with a worse effect in BD, with possible disruption of brain barriers permeability and an opposite effect of tightening and central segregation in MDD. Further analyses are needed to better elucidate this neurobiological mechanisms across mood disorders.

**Disclosure of Interest:**

None Declared

